# Role of Three-Dimensional Rotational Angiography in the Treatment of Spinal Dural Arteriovenous Fistulas

**DOI:** 10.7759/cureus.1932

**Published:** 2017-12-10

**Authors:** Yigit Ozpeynirci, Bernd Schmitz, Melanie Schick, Ralph Konig

**Affiliations:** 1 Neuroradiology, Ulm University; 2 Department of Radiology, Ulm University; 3 Department of Neurosurgery, Ulm University

**Keywords:** spinal, dural, avf, 3d-ra, angiography

## Abstract

Spinal dural arteriovenous fistulas (AVFs), being the most commonly encountered spinal vascular malformations, result in considerable morbidity with progressive spinal cord symptoms. A selective spinal digital subtraction angiography (DSA) is needed to confirm the diagnosis, to better evaluate the fine vascular structures and to plan therapy. With the introduction of three-dimensional rotational angiography (3D-RA), the information available for the treating physician has vastly increased. In the following article, we present a case series of four patients, in which the advantages of 3D-RA over the conventional biplane projections could be observed clearly.

## Introduction

Spinal dural arteriovenous fistulas (AVFs), being the most commonly encountered spinal vascular malformations and consisting of an anomalous connection between the radiculomedullary artery and the radicular vein inside the dura mater of the spinal nerve root, result in considerable morbidity with progressive spinal cord symptoms. The diagnosis is mainly based on magnetic resonance (MR) imaging, and a selective spinal digital subtraction angiography (DSA) usually follows to confirm the diagnosis, to better evaluate the fine vascular structures and to plan therapy [1]. With the introduction of three-dimensional rotational angiography (3D-RA), the information available for the treating physician has vastly increased. In the following article, we present a case series of four patients, in which the advantages of 3D-RA over the conventional biplane projections could clearly be observed.

## Case presentation

Four patients presented to our institution for selective DSA and treatment of spinal dural AVF between November 2014 and April 2015. The patients had variable degrees of spinal symptoms ranging, from lower back pain with minor gait difficulty to bladder and bowel incontinence. The Siemens Artis zee biplane angiography (Siemens, Erlangen, Germany) was used for all procedures. All patients were totally conscious during the procedures. All conventional DSA data were acquired with a 1024 x 1024-pixel matrix system. Biplane studies were performed for the feeding artery injections.

Data were acquired in a 960 x 960 matrix. A five-second acquisition protocol while holding breath was performed through a 200° rotation of the C-arm. Following a native rotational run, a hand injection of the artery of interest was performed with the Ultravist 300 mgI/mL nonionic contrast medium (Bayer, Leverkusen, Germany). This 133-image study was then transferred to a Siemens workstation in native format and processed by neuroradiology technicians with the assistance of the operating interventionalists. Multiplanar reconstructions of images (MPR), including maximum intensity projection (MIP) images and volume-rendered displays (VRD), were generated to demonstrate the anatomic relationships of the fine vascular structures. The reconstructed images along with the conventional subtraction angiograms were then discussed by the operating interventionalists and the vascular neurosurgeons with an emphasis on the treatment.

All fistulas were successfully occluded. According to the institutional protocol, complete treatment was confirmed by re-DSA within seven days after surgery.

Due to the exact localization of the fistulas, the surgical approach could be limited. Either total or partial hemilaminectomy was performed to access the fistulas. There was no need for extensive spinal approaches or spinal instrumentation. This was made possible by the high-resolution 3D-RA dataset, which provided exact guidance for the neurosurgeon. No surgical complications were seen, which caused a prolongation of hospital stay. A control-MRI in the sixth month after surgery showed different levels of regression of prior findings of venous stasis and myelopathy among the patients. A clinical examination revealed partial to full recovery, depending on the severity of the prior status of the patient.

In all of our patients, the 3D-RA could delineate the abnormal vascular anatomy in a very detailed manner. The site of the fistula could be clearly defined as well as the feeding artery and its connections with the surrounding normal vessels. The technique was easy to perform with a high level of patient compliance.

**Figure 1:**
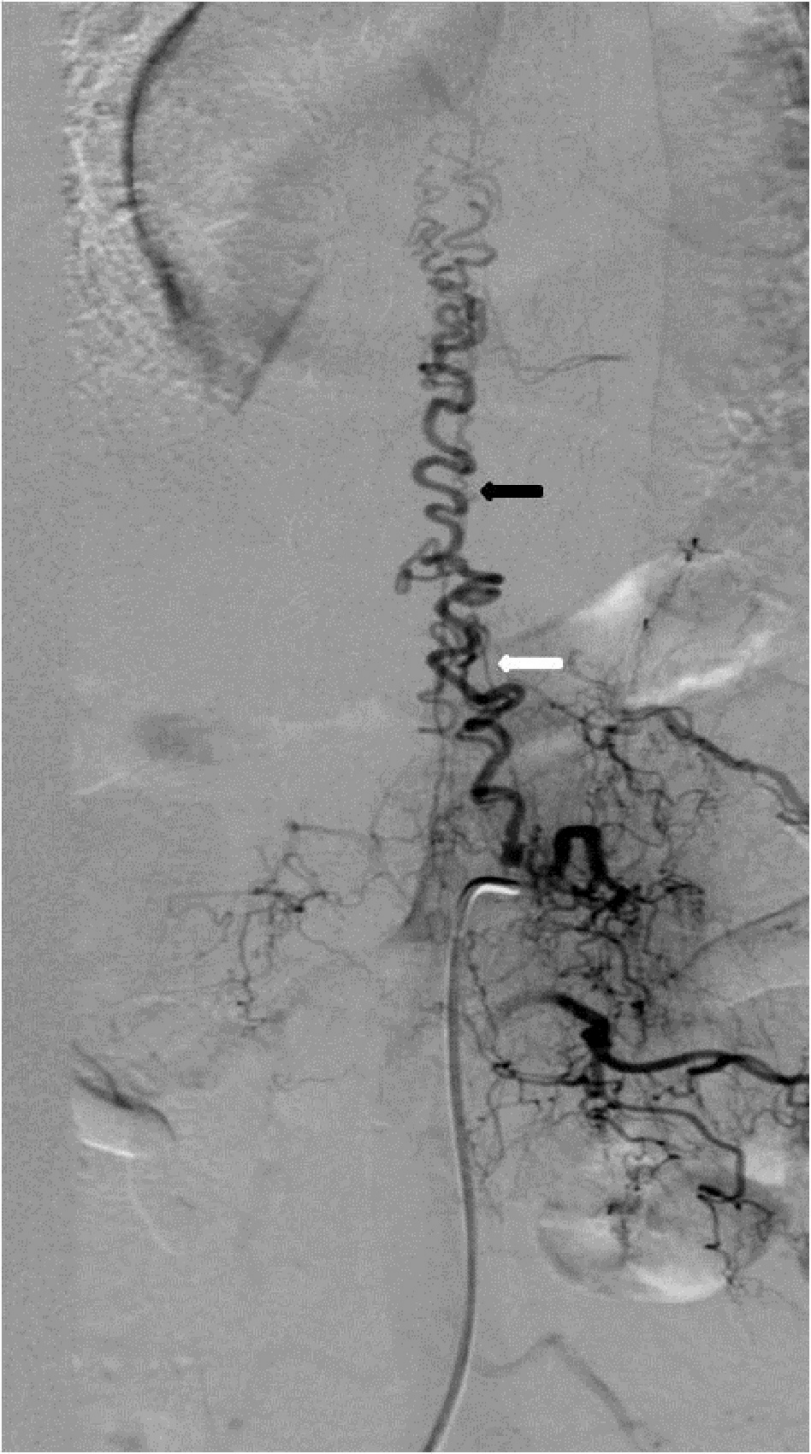
Case 1 The AP view showing a spinal dural AVF supplied by the left L1 radicular artery and opacification of the great radicular artery of Adamkiewicz (white arrow) and the draining veins (black arrow). AP: anteroposterior; AVF: arteriovenous fistula

**Figure 2:**
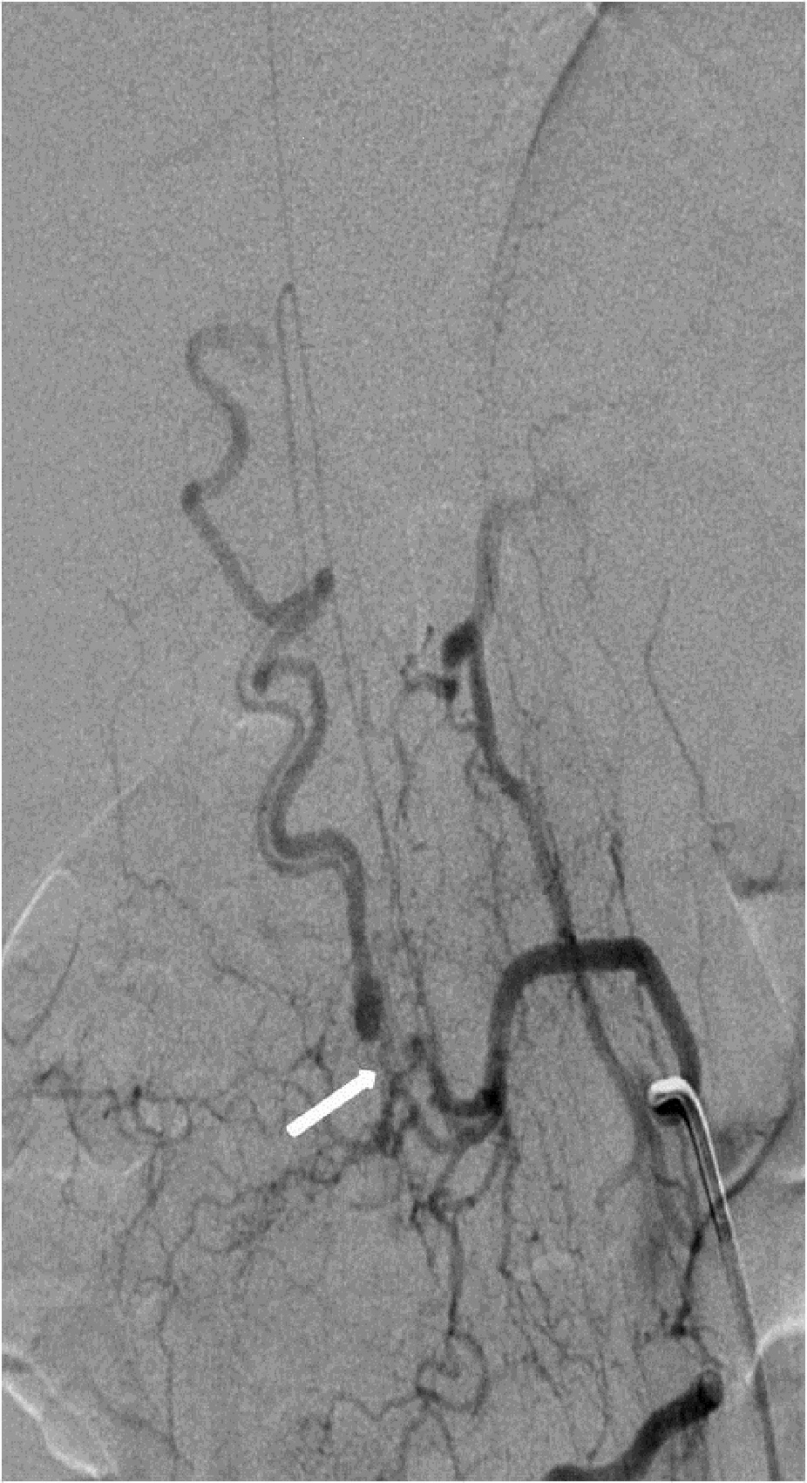
Case 1 Even the best possible oblique projection is not sufficient to demonstrate the exact origin of the great radicular artery. Arrow points to the possible site of the fistula.

**Figure 3:**
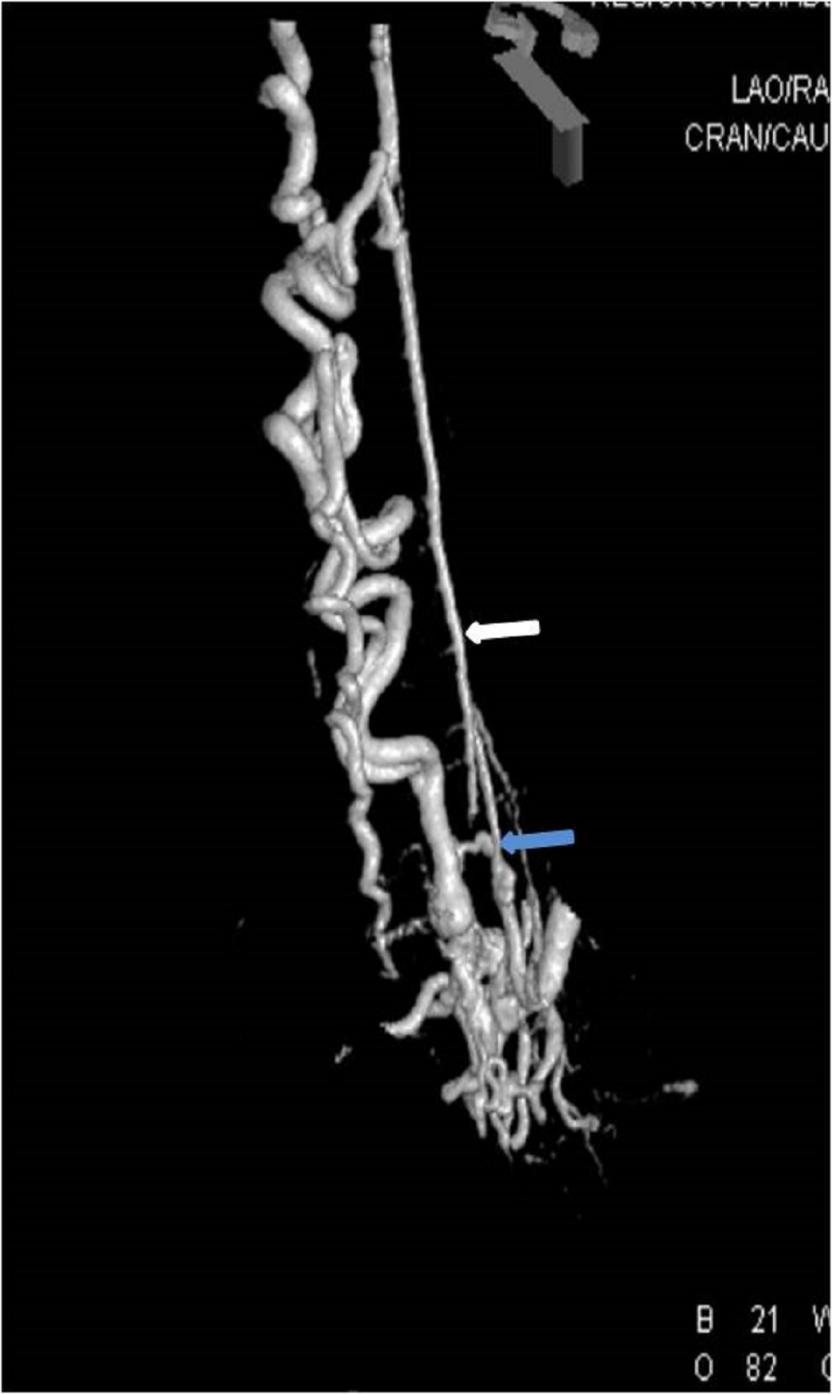
Case 1 Volume-rendered image easily delineates the vascular anatomy with the artery of Adamkiewicz (white arrow) originating just proximal to the fistula (blue arrow).

**Figure 4:**
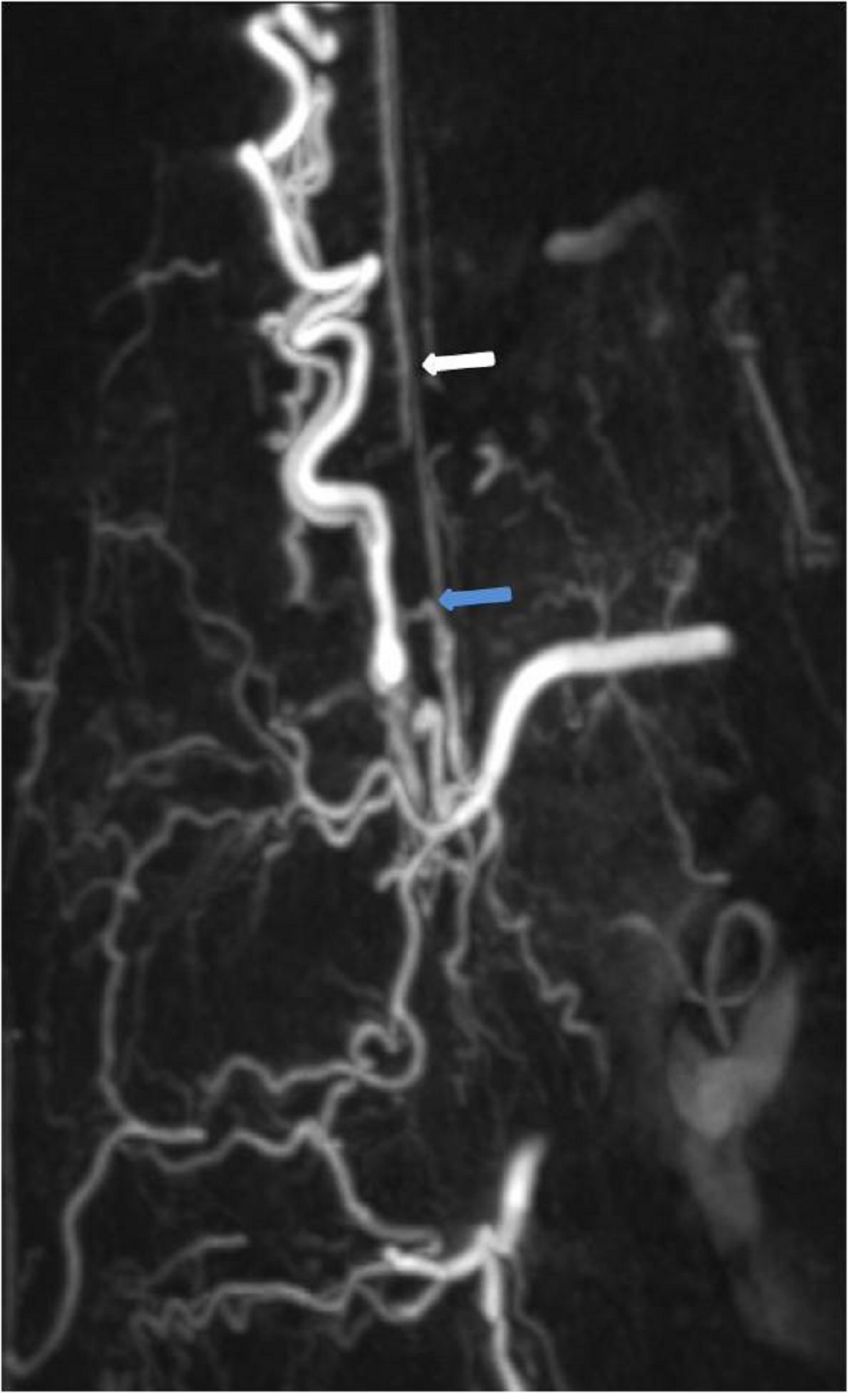
Case 1 MIP image easily delineates the vascular anatomy with the artery of Adamkiewicz (white arrow) originating just proximal to the fistula (blue arrow). MIP: maximum intensity projection

Especially in two patients, the advantages of the 3D-RA could be clearly appreciated. In the first patient, the conventional biplane DSA showed a spinal dural AVF supplied by the left L1 radicular artery. The segmental artery injection at this level also demonstrated contrast enhancement of the great radicular artery of Adamkiewicz with its typical hairpin turn. On multiple projections, the fistula was illustrated but the relationship between the origin of the artery of Adamkiewicz and the feeding artery of the fistula remained unclear. The 3D images could clearly depict the vascular anatomy with the artery of Adamkiewicz originating just proximal to the fistula (Figures 1-4). Consequently, superselective catheterization of the feeding pedicle in order to better understand the anatomy could be spared. Given the fact that no sufficient safe distance existed, which would allow a reflux of the embolization material proximal to the fistula, it was decided to treat the fistula surgically. It was successfully treated via a hemilaminectomy, as confirmed by a postoperative angiography (not shown).

**Figure 5:**
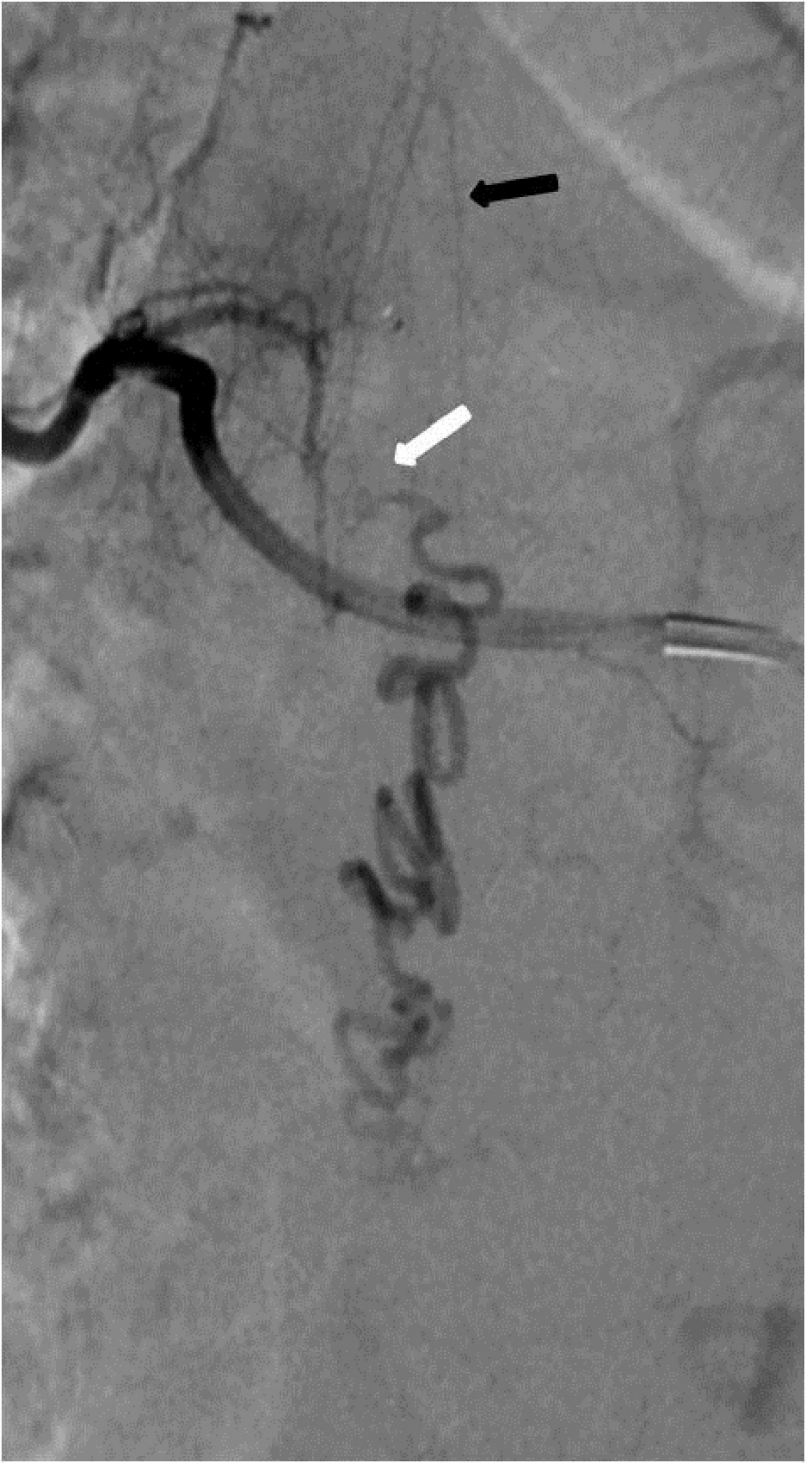
Case 2 DSA AP view demonstrates the fistula (white arrow) supplied by the left D6 segmental branch. A thin artery (black arrow), which is a contributor to the anterior spinal arterial circulation, seems to be originating just from the level of the fistula. DSA: digital subtraction angiography; AP: anteroposterior

**Figure 6:**
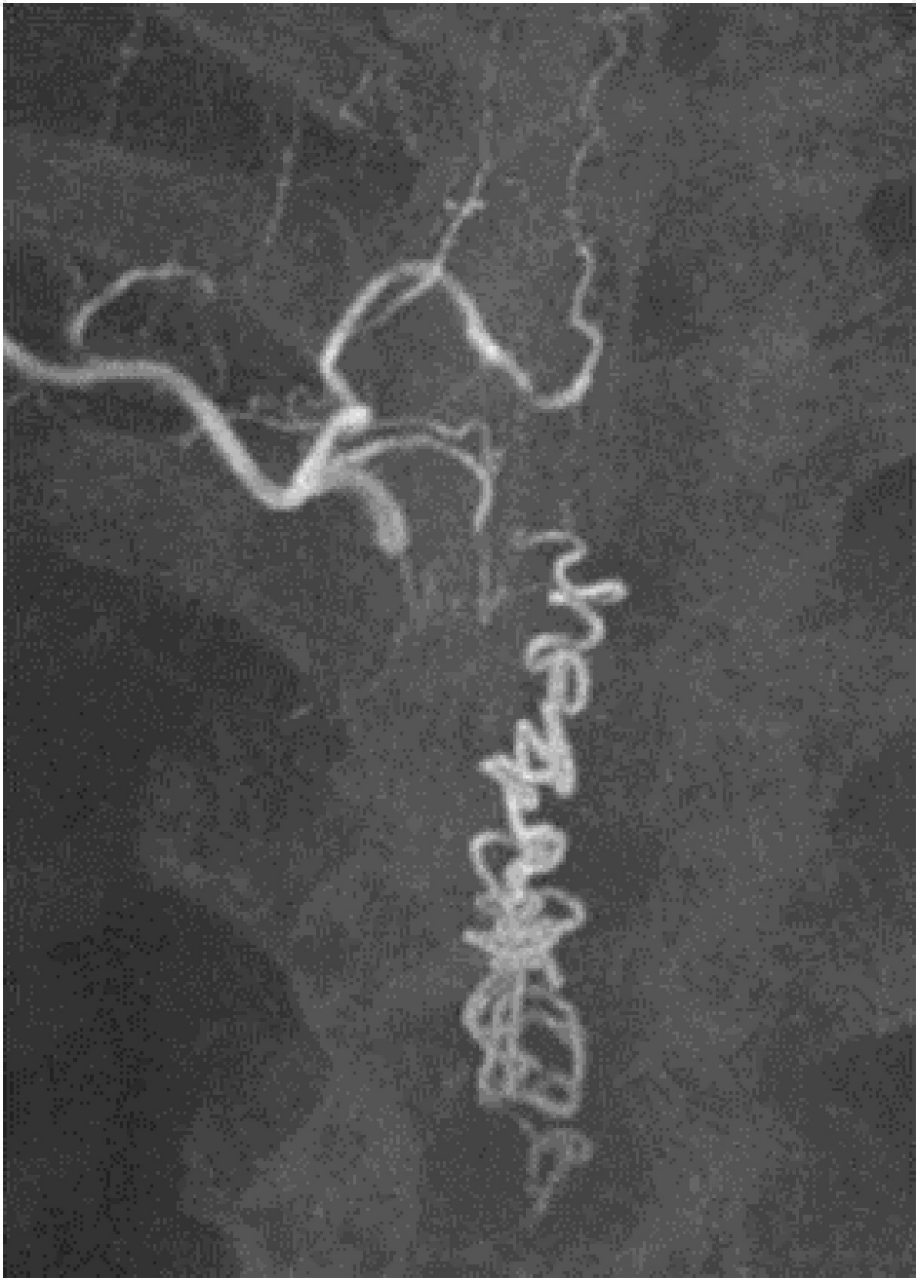
Case 2 MIP image shows the fistula nicely. MIP: maximum intensity projection

**Figure 7:**
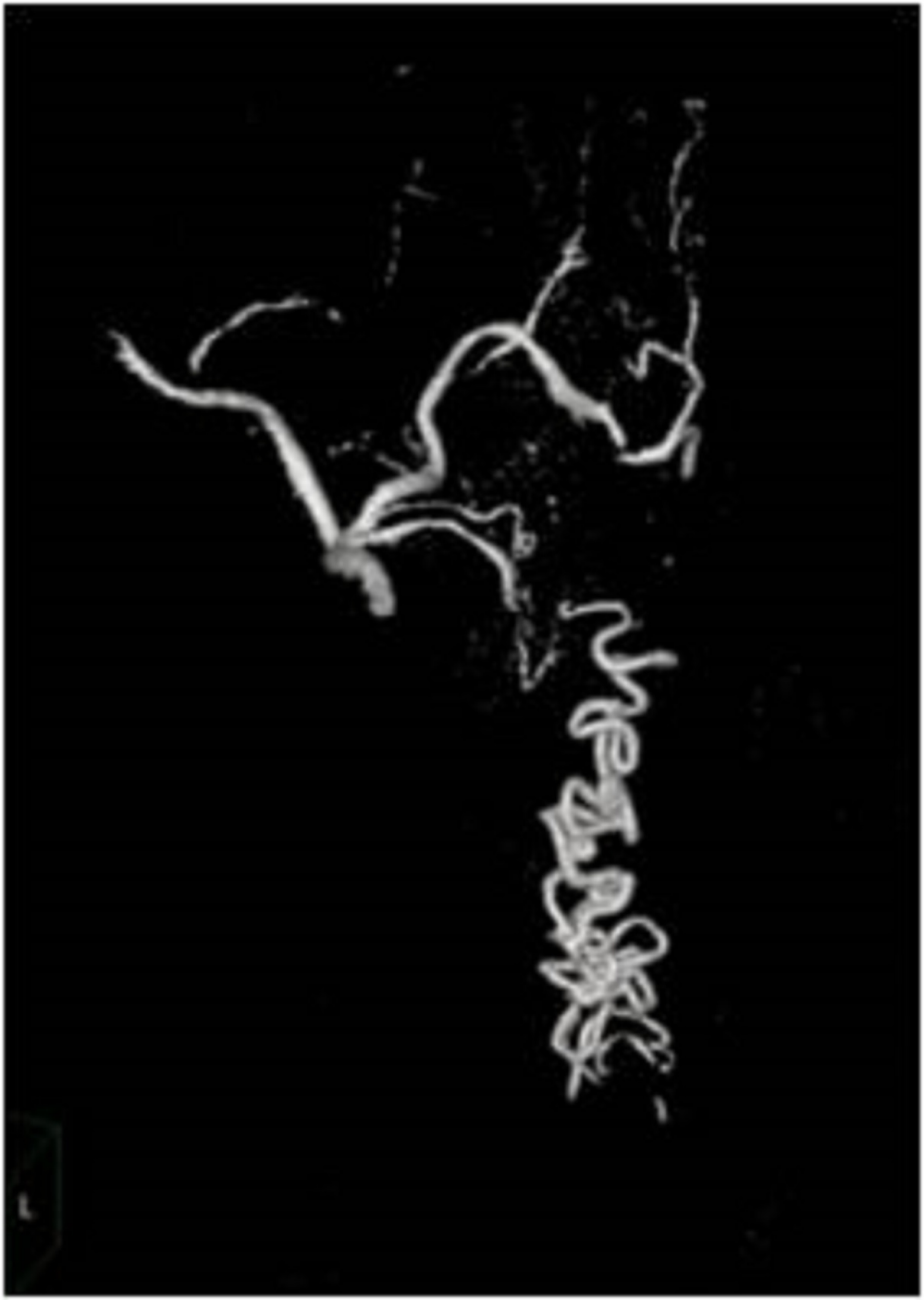
Case 2 Volume-rendered image shows the fistula nicely.

**Figure 8:**
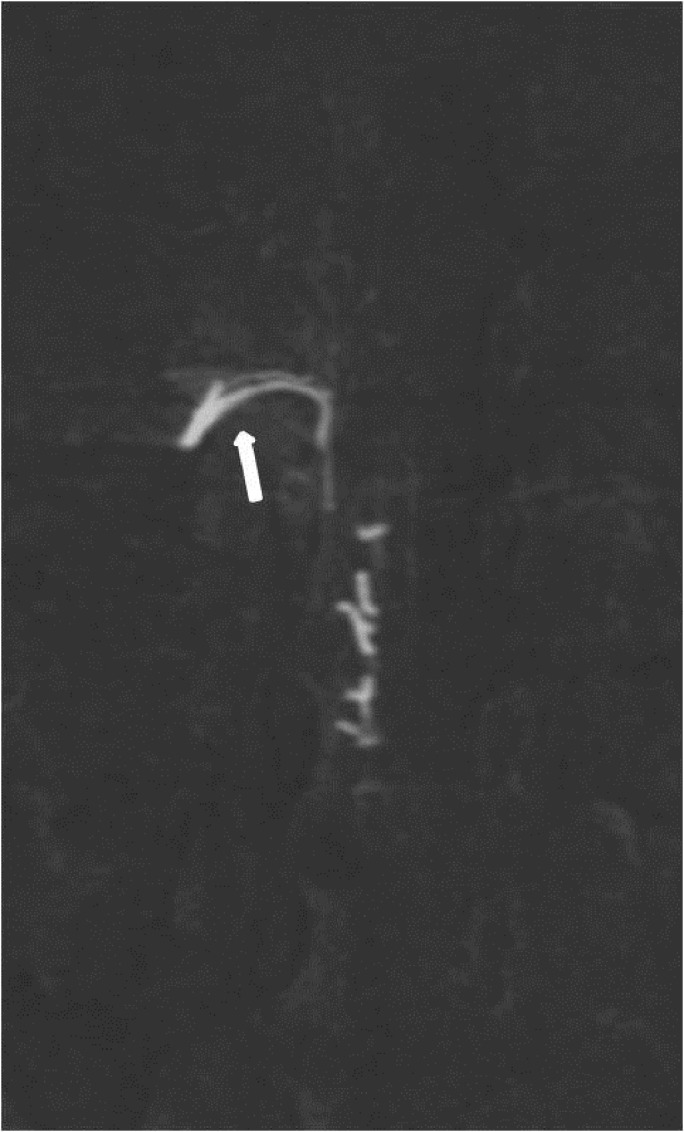
Case 2 MIP image depicts the origin of this thin artery, which originates from a much more proximal point (arrow) than the fistula but still from the feeding artery. MIP: maximum intensity projection

In the other patient with the spinal dural AVF supplied by the left D6 branch, the conventional biplane series showed a thin artery originating just from the level of the fistula, which seemed to be a contributor to the anterior spinal arterial circulation. The 3D images delineated that the artery originated from a much more proximal point than the fistula but still from the feeding artery (Figures 5-8). This information gained from the 3D images again made a selective catheterization unnecessary, which could have prolonged the angiography time and led to complications. This patient was also treated surgically.

**Figure 9:**
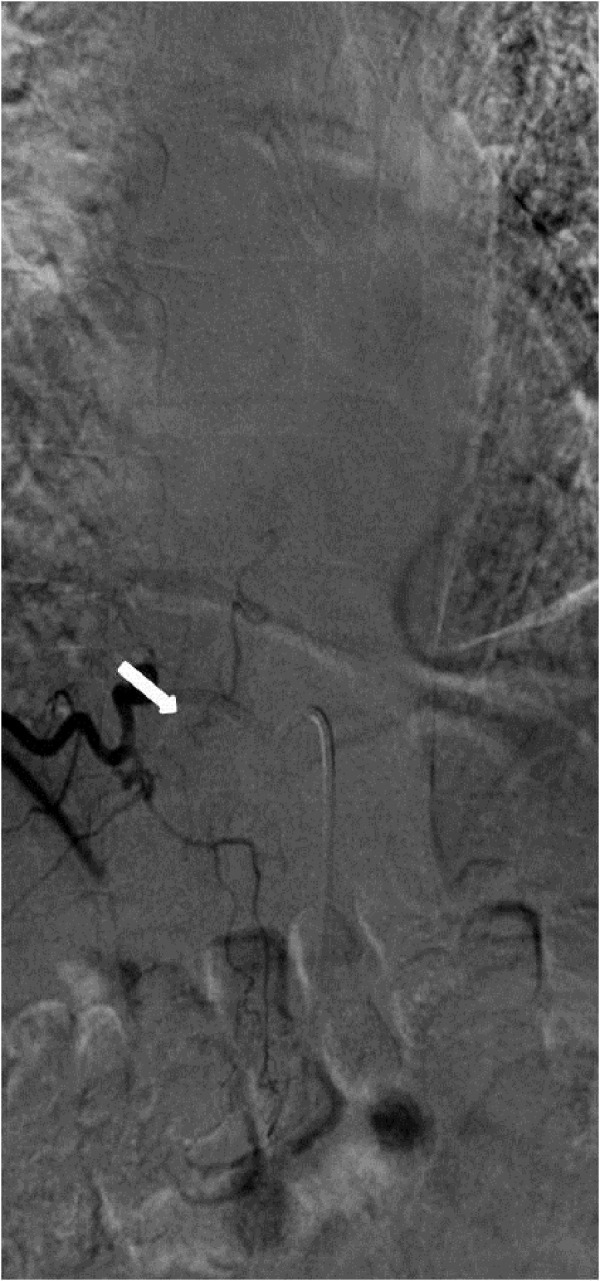
Case 3 Spinal dural AVF demonstrated more in detail on the 3D-RA than on the conventional DSA. The arrow points to the fistula. The complexity of the lesion can be better appreciated on the 3D images. AVF: arteriovenous fistulas; 3D-RA: three-dimensional rotational angiography; DSA: digital subtraction angiography

**Figure 10:**
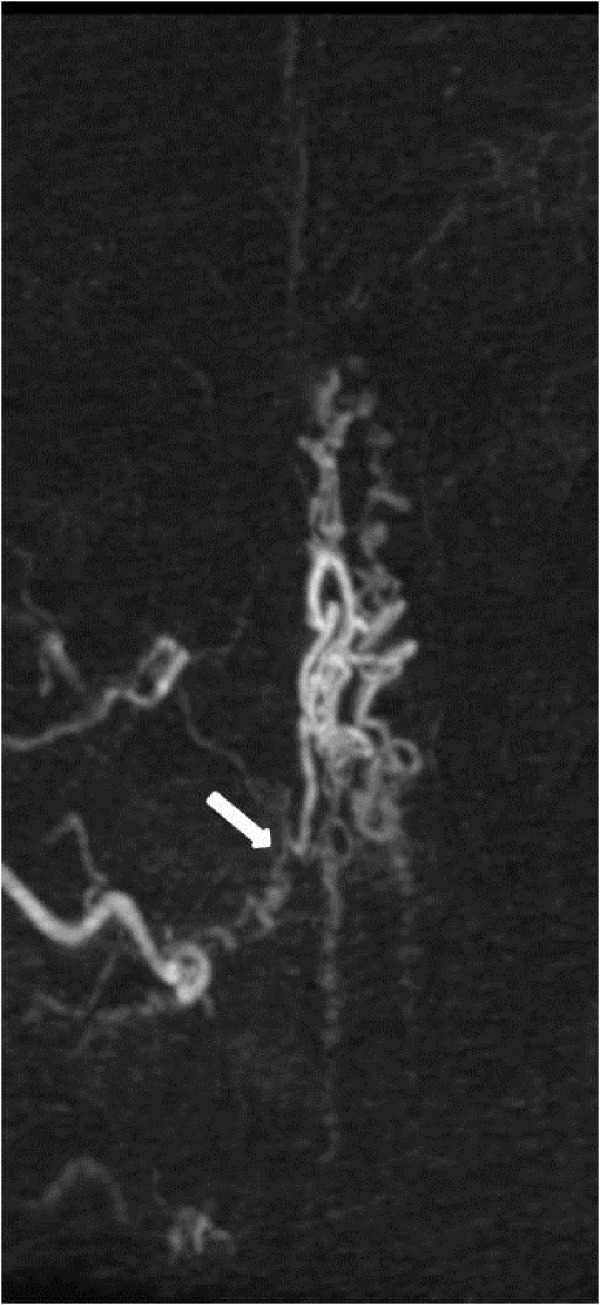
Case 3 Spinal dural AVF demonstrated more in detail on the 3D-RA than on the conventional DSA. The arrow points to the fistula. The complexity of the lesion can be better appreciated on the 3D images. 3D-RA: three-dimensional rotational angiography; DSA: digital subtraction angiography

**Figure 11:**
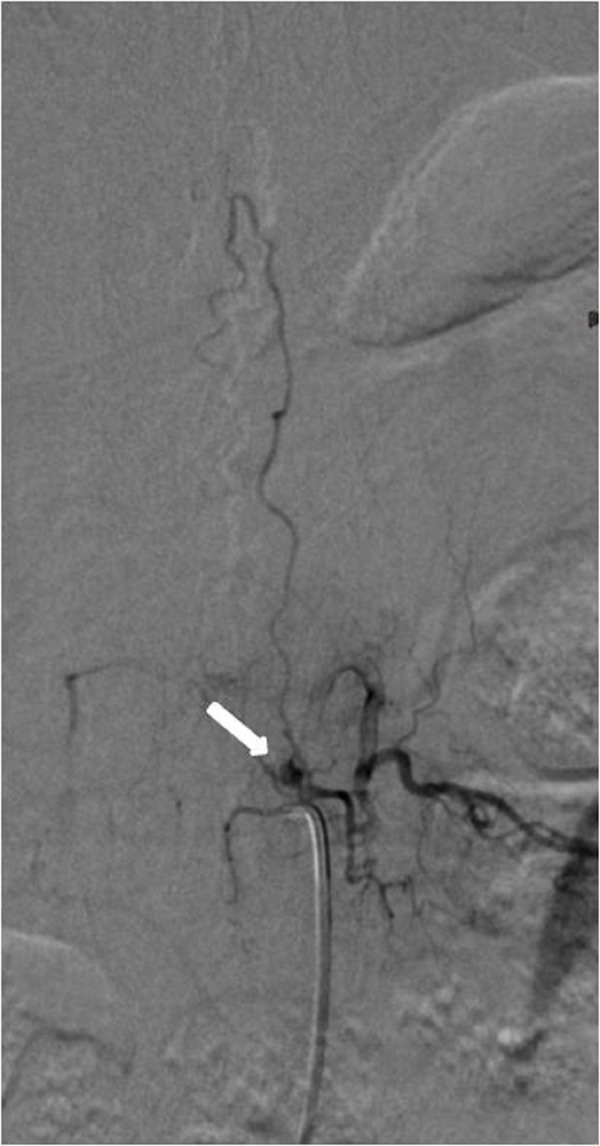
Case 4 Spinal dural AVF demonstrated on the conventional DSA. The arrow points to the fistula. AVF: arteriovenous fistula; DSA: digital subtraction angiography

**Figure 12:**
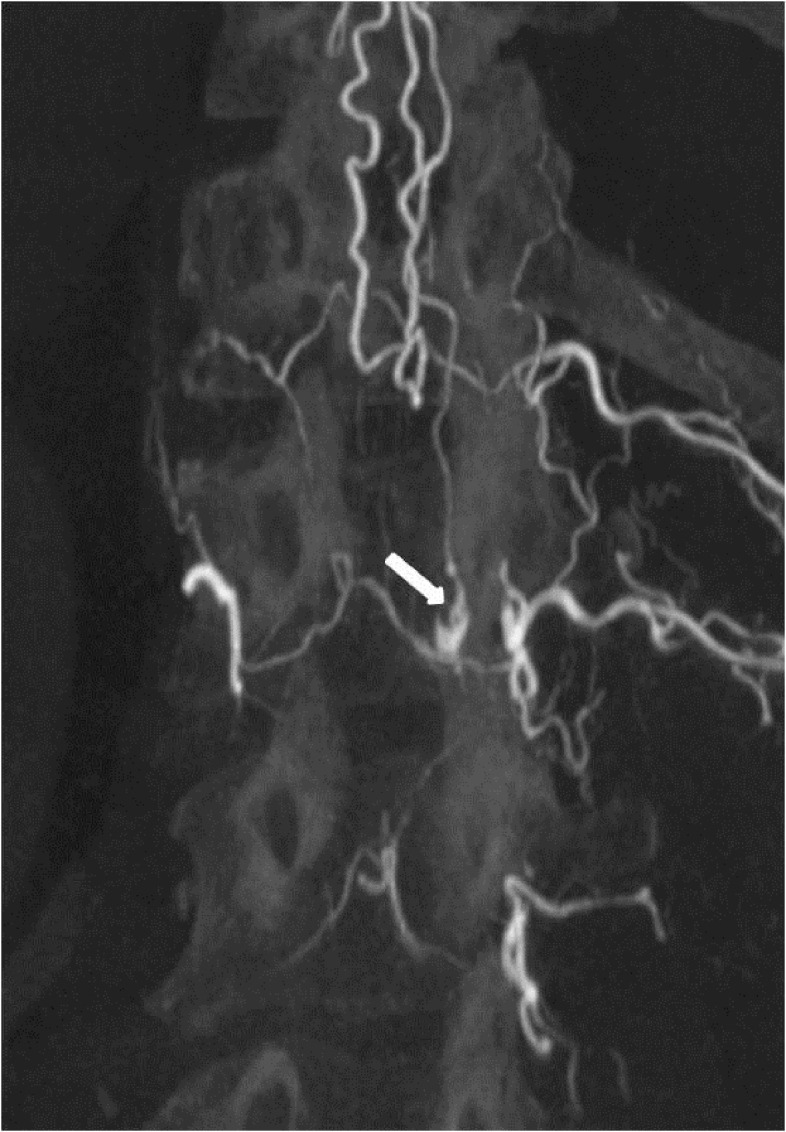
Case 4 Spinal dural AVF demonstrated on the 3D-RA. The arrow points to the fistula. The complexity of the lesion can be better appreciated on the 3D images. AVF: arteriovenous fistula; 3D-RA: three-dimensional rotational angiography

The other patients had simpler fistulas, which could also be very well depicted using the 3D-RA and then successfully treated surgically (Figures 9-12).

## Discussion

In several case studies, which employed 3D-RA to demonstrate the vasculature of the spinal cord, 3D-RA provided very useful information on the spinal vascular anatomy and the relationship between the vessels and the spinal cord as well as the surrounding bony structures. Some of those studies included all spinal vascular malformations and spinal tumors as well. The main advantages of this technique are summarized as follows: differentiating intramedullary from perimedullary surface vascular lesions; detecting arterial, nidal, or venous aneurysms; selecting a working angle for manipulating the microcatheter and for glue injection; detecting an intraoperative subarachnoid hemorrhage in a timely manner during endovascular treatment of spinal vascular diseases [2-7].

In our series, this technique provided detailed information for either embolization or microsurgical occlusion. 3D-RA is able to clarify anatomic relationships for the feeding vessel, especially in areas where the demonstration of the lesion is difficult with conventional multiple projection imaging, e.g. lower thoracic and lumbar lesions.

In all of our patients, 3D-RA could depict the vascular anatomy very well. The side of the fistula could be clearly defined as well as the feeding artery and its connections with the surrounding normal vessels. The technique was easy to perform with a high level of patient compliance.

## Conclusions

3D-RA has proven itself to be an effective method in the diagnostic workup of spinal dural AVF. A variety of valuable information can be gained from a single rotational view that would have otherwise required multiple oblique views or superselective catheterization. 3D angiography provided better anatomic detail, enabling very precise and successful neurosurgical procedures to treat spinal vascular malformations.
